# Investigation of Penetration Performance of Zr-based Amorphous Alloy Liner Compared with Copper

**DOI:** 10.3390/ma13040912

**Published:** 2020-02-19

**Authors:** Ping Cui, Deshi Wang, Dongmei Shi, Xinbao Gao, Jingqing Xu, Jianwei Zhen

**Affiliations:** 1 College of Weaponry Engineering, Naval University of Engineering, Wuhan 430033, China; 2 Department of Ammunition Engineering of Shijiazhuang Campus, Army Engineering University of PLA, Shijiazhuang 050003, China; 18622606495@163.com (D.S.);; 3 Military Key Laboratory for Ammunition Support and Safety Evaluation, Shijiazhuang 050003, China

**Keywords:** stand-off distance, energetic liner, Zr-based amorphous alloy, depth of penetration (DOP), experimental investigation

## Abstract

Zr-based amorphous alloy is a new type of metastable energetic material, which has been exploringly used to design shaped charge (SC) liners by scholars of the military industry. In order to know well how the stand-off distance influences jet penetration performance of liners made by such energetic materials against metal targets, SC static explosion tests were conducted under the same initiation and target conditions but different stand-off distances compared with copper liners. Test results indicate that the jet depth of penetration (DOP) of Zr-based amorphous alloy liners firstly increases slowly and then decreases sharply as the stand-off becomes larger. The optimum stand-off distance is 3.5 times of charge diameter (CD) and the corresponding maximum DOP is about 2.68 CD against the 45# steel plate. The perforation area varies with the stand-off distance. It reaches the maximum when the stand-off is 3.5 CD and the corresponding perforation diameter is about 42mm, also the penetration hole is nearly circular. The jet DOP of Zr-based amorphous alloy liner is smaller than that of copper liner’s while the perforation area is the opposite. The former DOP is about 55.7% of the latter and the former perforation area is about 2.8 times of latter when the stand-off distance is 3.5 CD.

## 1. Introduction

The depth of penetration (DOP) of the shaped charge jet to the target plate is related to the stand-off distance between the shaped charge (SC) and target, which is called the optimum or favorable stand-off when the peak penetration occurs [1]. It is also called “focal length” in some references. The SC penetration ability varies hugely with stand-off, even the explosive charge, liners, targets, initiation mode and other conditions are the same. In general, the jet is not formed completely and its penetration ability is limited when the stand-off distance is too small. The conflict between jet stretch and jet fracture occurs when the stand-off is too high. For commonly used liners, the optimum stand-off is about 2–3 times charge diameter (CD). The penetration effect will be reduced when the stand-off distance is less than or larger than the optimal value. The study of the stand-off influence on the penetration efficiency presents important references for the design of the shaped charge warhead, and ultimately determines whether the warhead can play the maximum damage efficiency.

Scholars have carried out a lot of research about the stand-off influence on the jet penetration performance of non-energetic single element metals or composite liners [2,3,4,5,6,7,8,9,10,11], which will not be described in this article. The application of energetic materials (also known as active materials and reactive materials) on liners makes the SC have not only the high-efficiency penetration, but also the aftereffect of combustion, detonation and heat release, further enriching the damage mode of antiarmor ammunition to the target. The damage form of this kind of liners to the target is quite different from that of ordinary liners. Baker and Daniels investigated the reactive jet formation behavior by using an X-ray pulse and experimentally analyzed the effects of a stand-off on penetration performance [12]. Wang et al. [13] carried out a series of experiments at stand-offs of 1.0, 2.0 and 2.5 CD to determine the influence of PTFE/Al reactive liner thickness on the penetration performance of the shaped charge. Guo et al. [14] fabricated a novel high-density reactive material liner based on the PTFE matrix and investigated the corresponding penetration performance. The static experimental results demonstrated that the penetration depth of this high-density reactive jet increased firstly and then decreased by increasing the stand-off. Zhang et al. [15] designed the active material liner of aluminum/nickel metal system based on the large perforation demand of petroleum perforating bullet. The static explosion test conducted at a fixed stand-off of 60mm showed that adding a certain proportion of reactive metals can improve the aftereffect ability of the liner jet and increase the perforation aperture. Liu et al. [16] used LS-DYNA software to simulate and verify the jet characteristics and damage performance of PTFE/Al system active material liner. The results showed that the armor penetrating performance was the best at the stand-off of 1.0–1.5 CD, and the maximum DOP could reach 65mm. Sun et al. [17] carried out penetration experiments of nickel-aluminum (Ni-Al) and copper-nickel-aluminum (Cu-Ni-Al)-shaped charge liners into 45# steel at a fixed stand-off distance. The results showed the continued exotherm of Ni-Al reactive jet when it was fired into the target. The addition of Cu could not only increase the average crater size, but also raise the average penetration depth by 42%.

As a new type of energetic material, Zr-based amorphous alloy has high strength, high hardness and good energy release characteristics. The SC jet formed by this kind of metastable state material can produce violent chemical reaction during or after penetrating the target plate, release a lot of chemical energy, and eventually achieve much higher efficient damage. Walters et al. [18,19] from the US Army Research Laboratory studied the jet forming and penetration behavior from a shaped charge liner of Vitreloy 106, which is a Zr-based amorphous alloy with the atomic composition of Zr_57_Nb_5_Cu_15.4_Ni_12.6_Al_10_. The results showed that this kind of liner has good suitability as a possible jetting material. Among all the three static explosion tests, two are free flight tests and the other one is a small stand-off penetration test, which got a total penetration depth of 232.6mm. However, Walters did not further study the penetration ability of the Zr-based amorphous alloy liner under other series of stand-offs. On the basis of experimental investigation on mechanical properties of bulk amorphous alloy materials [20,21], Shi et al. have carried out simulation research on the jet forming characteristics and armor penetration ability of the ZrCuNiAlAg amorphous alloy materials by using AUTODYN numerical simulation software [22]. Zhang et al. conducted theoretical and experimental study on the impact compression behavior of ZrTiNiCuBe amorphous alloy [23], failure behavior and energy release of ZrCuNiAlAg amorphous alloy under dynamic compression [24], and shock energy release characteristics [25]. They also studied penetration and energy release characteristics of the W/ZrNiAlCu metastable reactive alloy composite fragment against the RHA target [26]. Shang et al. summarized the shock energy release reactions, reviewed the damage research of the Zr-based amorphous alloy as shaped charge liners and measured the combustion heat of Zr_66_Cu_24_Al_10_ [27]. None of the above work involves the experimental research of jet forming and penetration performance of the Zr-based amorphous alloy liner, and related influencing rules of the stand-off distance.

To sum up, Zr-based amorphous alloy materials have been widely used in new energetic warheads and have attracted the attention of scholars in the field of military industry from all over the world. In this paper, the jet penetration performance of Zr-based amorphous alloy liner to metal targets under different stand-off distances is fully studied through the comparative test with copper liners under the same conditions, so as to accurately grasp the damage characteristics of this kind of energetic liner.

## 2. Experimental Procedure

**Figure 1:**
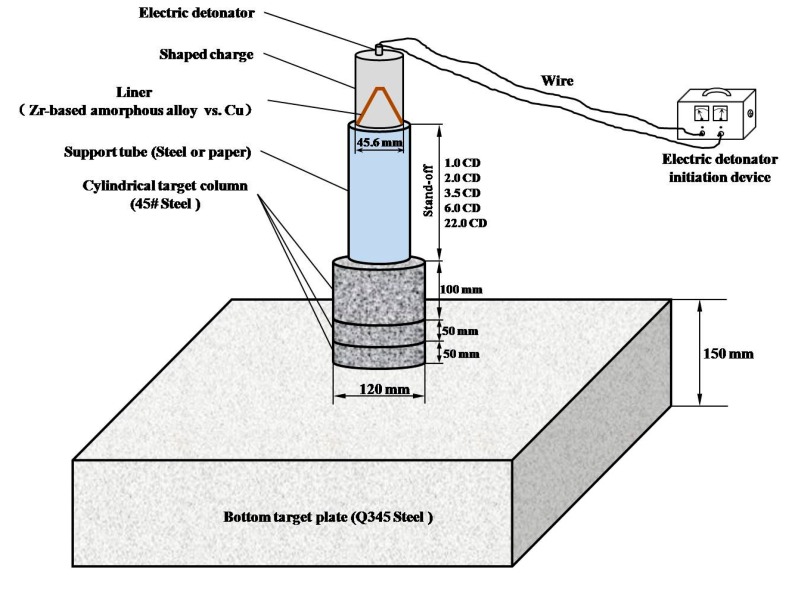
Diagram of the test device.

**Figure 2:**
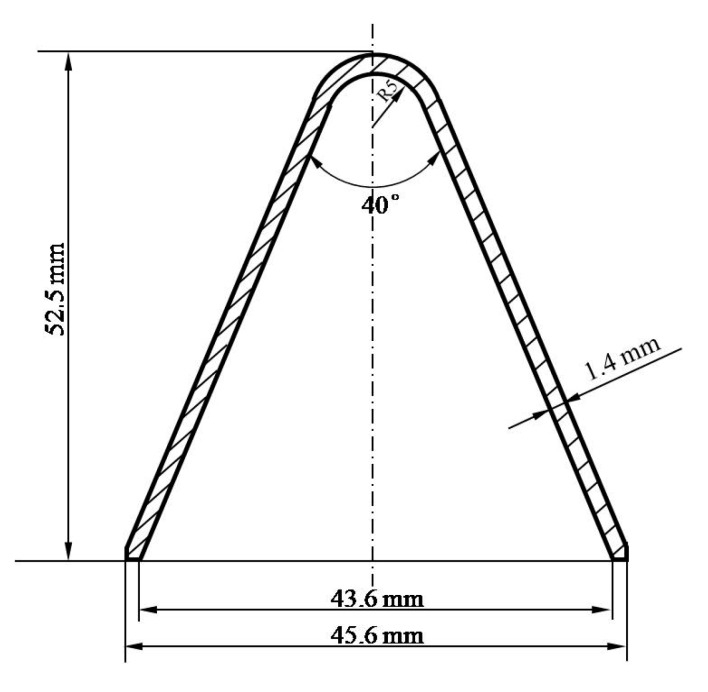
Sketch of the conical liner.

**Figure 3:**
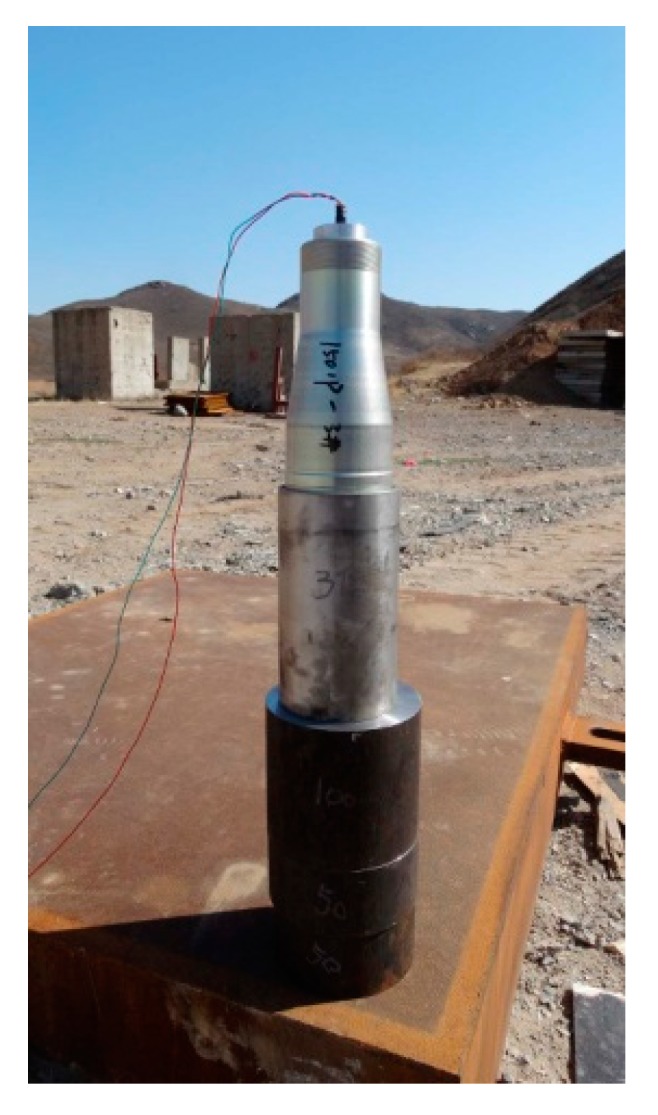
Layout of the testing site (stand-off 3.5 CD).

The typical device of the static explosion test for the penetration performance of jet into the metal target plate is usually composed of a shaped charge, a support tube controlling the stand-off distance and several metal blocks with specific materials and thickness. This method has been widely used by scholars [28,29,30]. The test device configuration diagram in this paper is shown in Figure 1. JH-2 (Hanfeng Changbai Technology Co., Ltd, Qinhuangdao, China) is used for both the front and rear explosive charge. One CD was defined to be 45.6 mm. Liners are bonded to the shaped charge after the front explosive charge been pressed. The energetic liner was made by vacuum liquid near-clean die-casting forming method and its material was a Zr-based amorphous alloy (Institute of Metal Research, Shenyang, China) with an intended composition of Zr_63.5_Cu_12_Ni_10_Al_12.5_Ag_2_ (Institute of Metal Research, Shenyang, China)_,_ given in atomic percentages. Figure 2 shows the sketch of the conical liner. The angle of the cone was 40°, the thickness was 1.4 mm, the liner diameter was 45.6 mm and the height of the liner was 52.5mm. The material of the target plate was 45# steel and Q345 steel. The target plate was composed of three 45# steel cylindrical targets with a thickness of 100 mm, 50 mm and 50 mm respectively and Q345 steel base plate with a thickness of 150 mm stacked from top to bottom. After the preparation of the test site and target plate and ensuring the safety of the site, first place the supporting tube and the shaped charge warhead, then connect the detonating wire and install the electric detonator, finally connect the wire to the electric detonator and implement initiation. In order to study the effect of stand-off on the penetration effect of Zr-based amorphous alloy liner, some steel or paper supporting tubes with height of 1.0, 2.0, 3.5, 6.0 and 22.0 CD were used to control the stand-off distance. JH-2 explosive charge was detonated by electric detonators. The damage to the target plates caused by jets of such energetic liners were observed and recorded, including DOP and the entrance and exit size of potential perforations. Eight electric detonators were consumed in the test process. While the other conditions remain the same, Zr-based amorphous alloy liners are replaced by copper liners, which are widely used in current armor piercing equipment. Comparative tests with copper liners were carried out under the stand-off distance of 3.5, 6.0 and 22.0 CD. All the damage morphology of the target plates caused by copper jets were recorded to be compared with Zr-based amorphous alloy liners. Figure 3 shows the layout of the testing site when the stand-off distance was 3.5 CD.

## 3. Results and Discussion

**Figure 4:**
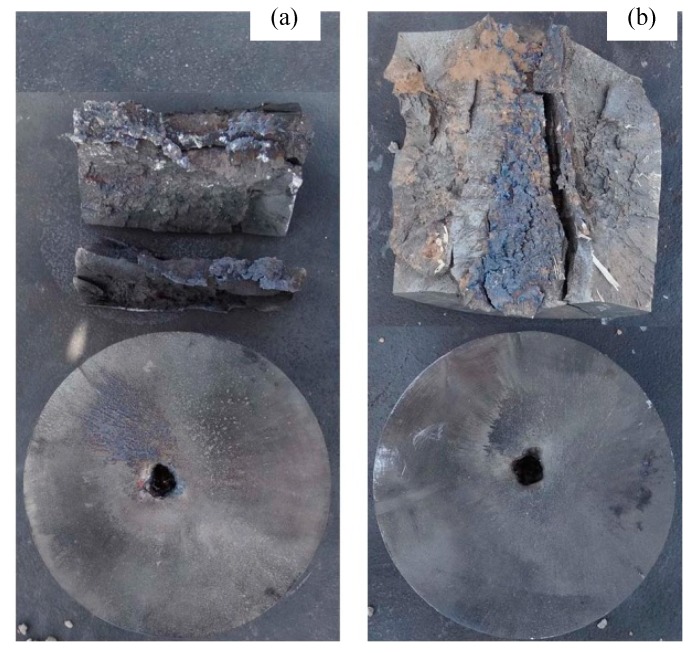
Part of the splintered blocks from the 1^st^ target column and pattern of penetration holes formed in the 2^nd^ target column. (**a**) Stand-off 1.0 CD and (**b**) stand-off 2.0 CD.

**Figure 5:**
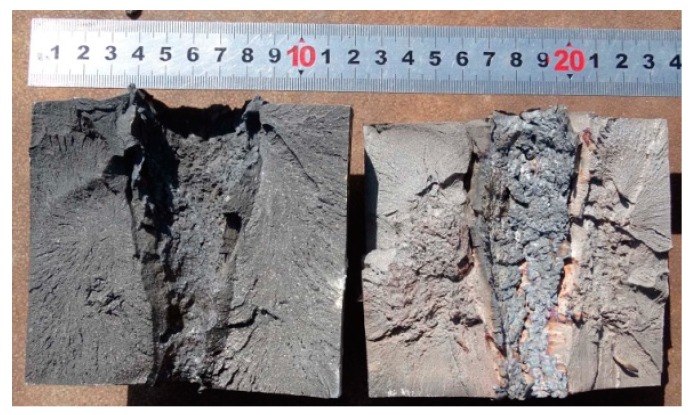
Splintered blocks from the 1^st^ target column (stand-off 3.5 CD).

**Figure 6:**
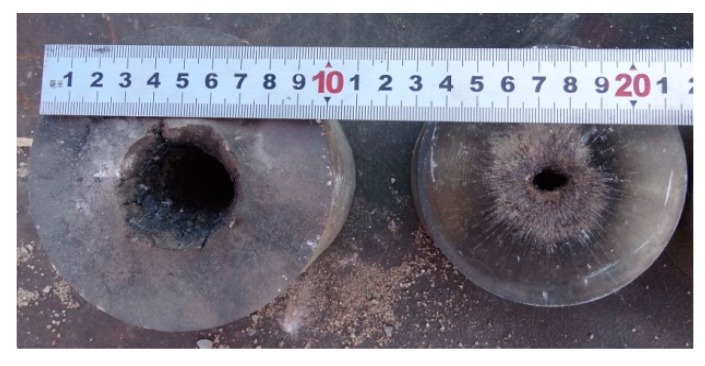
Pattern of penetration holes formed in the 1^st^ and the 2^nd^ target columns (stand-off 6.0 CD).

**Figure 7:**
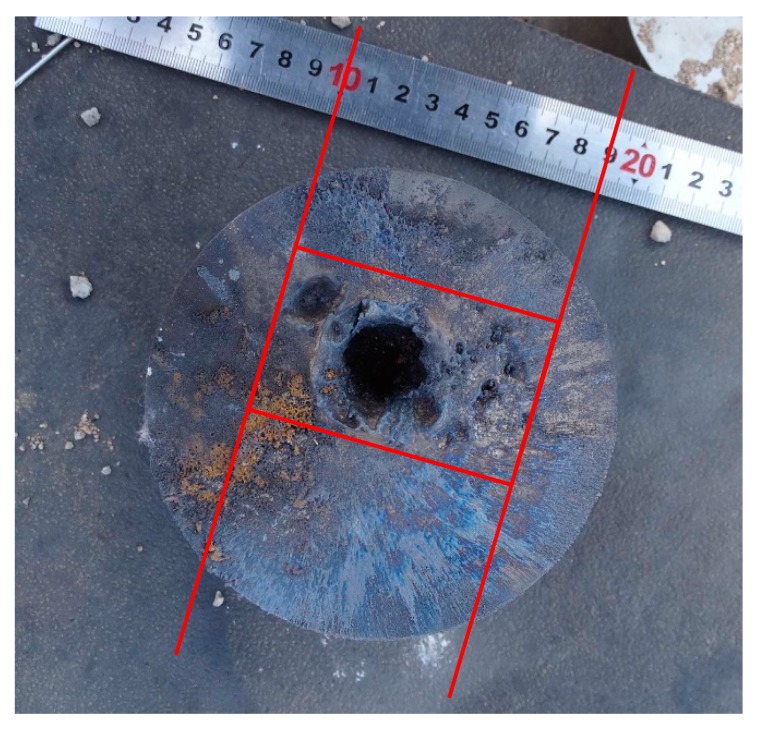
Pattern of penetration holes formed in the 1^st^ target column (stand-off 22.0 CD).

The jets formed by Zr-based amorphous alloy liner penetrated into the 2^nd^ target columns at most under each stand-off distance, while the 3^rd^ target column and the bottom target plate were not damaged in the whole test process. Only the 1^st^ target column was damaged when the stand-off distance was 22.0 CD. The 1^st^ target columns disintegrated or split into several pieces in the static explosion process when the stand-off distance was 1.0, 2.0 and 3.5 CD, and two of them could not be recovered when the stand-off distance was 1.0 and 2.0 CD. The static explosion test results of Zr-based amorphous alloy liner, such as the DOP and perforation size, are shown in Table 1. Figure 4 shows part of the splintered blocks recovered from the 1^st^ target columns and the perforation patterns of the 2^nd^ target columns at 1.0 and 2.0 CD respectively. Figure 5 shows the splintered blocks and the perforation internal morphology of the 1^st^ target column at 3.5 CD. Figure 6 shows the perforation morphology of the 1^st^ and the 2^nd^ target columns at 6.0 CD. Figure 7 shows the perforation morphology of the 1^st^ target column at 22.0 CD.

**Figure 8:**
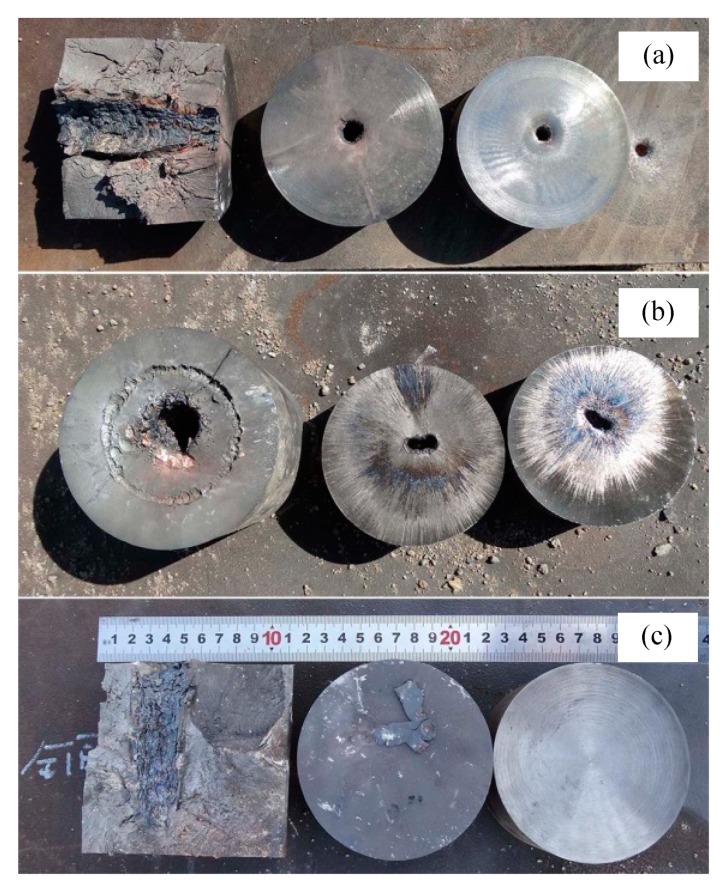
Damage to target plates produced by copper liner jet. (**a**) Stand-off 3.5 CD; (**b**) stand-off 6.0 CD and (**c**) stand-off 22.0 CD.

The comparison test of static explosion with a copper liner has been conducted and the results are shown in Table 2. Under the stand-off distance of 3.5 CD, the jet formed by copper liner penetrates three 200 mm thick cylindrical targets and formed a 19 mm deep perforation in the bottom target plate. In this process, the 1^st^ target column cracks into three pieces, and the perforation morphology in the other target columns and bottom target are shown in Figure 8a. Under the stand-off distance of 6.0 CD, the copper jet penetrated the 1^st^ and the 2^nd^ target columns, and formed a 33mm deep perforation in the 3^rd^ target column. The perforation morphology of each target column is shown in Figure 8b. The circular indentation around the perforation on the 1^st^ target column is formed by the steel tube during the static explosion. In addition, a certain degree of hole plugging was found in the 2^nd^ target column. When the stand-off distance was 22.0 CD, only perforation with a depth of 93 mm was formed in the 1^st^ target column, and no damage was found on the 2^nd^ and 3^rd^ target columns, as shown in Figure 8c.

### 3.1. DOP

**Figure 9:**
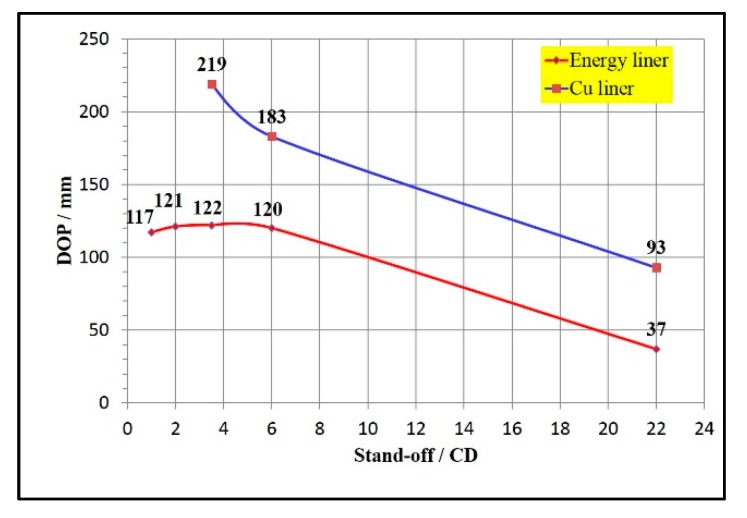
Depth of penetration (DOP) variation curve with a stand-off distance of the Zr-based amorphous alloy and copper liner.

In the case of the same explosive type, charge amount, initiation mode, target plate and other conditions, the DOP of Zr-based amorphous alloy liner increased slowly firstly and then decreased sharply with the increase of stand-off distance, and its change curve is shown in Figure 9. The most optimum stand-off distance was about 3.5 CD. At this stand-off, the DOP of the energetic liner to the 45# steel plate was 122 mm, and the maximum DOP was about 2.68 times of the charge diameter. It can also be seen from the curve that the change of DOP under the stand-off distances of 2.0, 3.5 and 6.0 CD was not obvious.

### 3.2. Perforation Area

**Figure 10:**
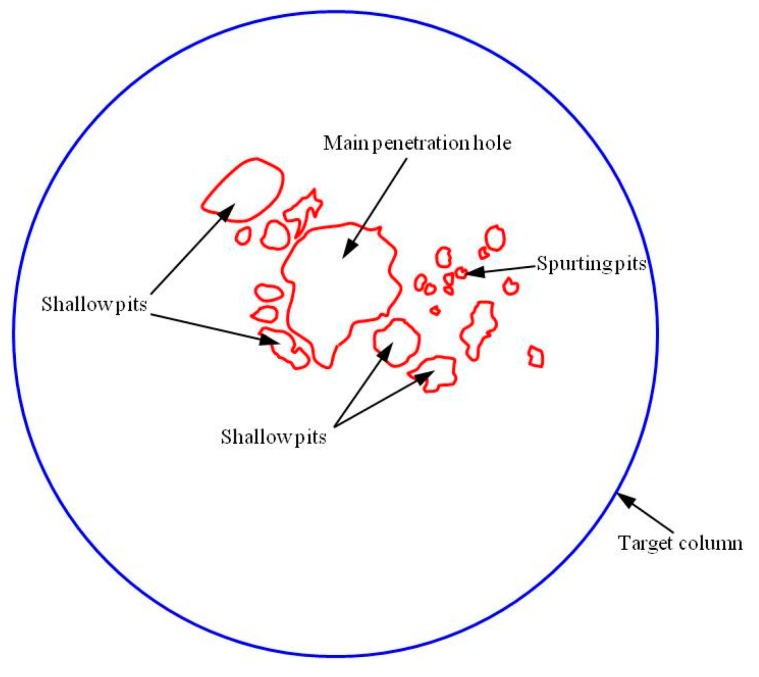
Schematic of penetration holes formed in the 1^st^ target column by the energetic liner (stand-off 22.0 CD).

**Figure 11:**
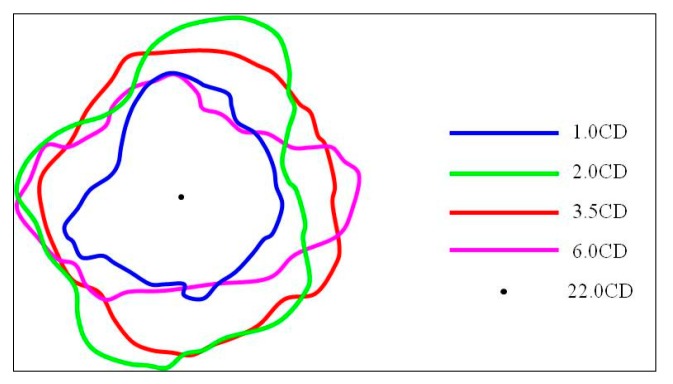
Profiles of entrance holes formed in the 2^nd^ target columns by energetic liners.

The perforation area produced by jets of Zr-based amorphous alloy liners varied with the stand-off distance. The 1^st^ target columns fragmented during the penetration when stand-off distance was 1.0 CD and 2.0 CD, therefore it was impossible to gather all the cracked targets to recover it into a whole target column. That means the dimension of the perforation entrance could not be gauged accurately under these two stand-off distances. From the test results of 3.5, 6.0 and 22.0 CD, it can be seen that the size of perforation entrance is the largest when the stand-off distance is 3.5 CD, and the diameter is about 42mm. When the stand-off distance is much higher (22.0 CD) than the optimum stand-off, the size of the perforation entrance is the smallest with a diameter of about 25 mm. However, from the perspective of the surface morphology of the target after penetration (as shown in Figure 7), in addition to a main perforation with a diameter of about 25 mm, there are four shallow pits with larger area and several sputtering pits with smaller area and depth (the perforation morphology and distribution diagram are shown in Figure 10), which indicate the presence of dispersion and particulation of the jet formed by Zr-based amorphous alloy liner during the long distance flight. If judging from the entrance size of the 2^nd^ target column, the perforation area was smaller when the stand-off distance was 1.0 CD and no perforation formed when the stand-off distance was 22.0 CD. Although the difference of perforation area was not obvious when the stand-off distance was 2.0, 3.5 and 6.0 CD, it could be seen that the perforation shape was closer to the circle when the stand-off distance was 3.5 CD, which indicates that the jet transverse divergence was the smallest under this condition. Figure 11 shows the comparison of the shapes of perforation entrances in the 2^nd^ target columns at the same scale under each stand-off distances.

### 3.3. Comparison with Copper Liner

Copper (Cu) was the best choice for liner material of shaped charge at present. About influence of the stand-off distance on the Cu liner penetration effect, there are lots of investigations in corresponding references, which was not the analyzing focus of this paper. We here made a comparison between the Cu liner and Zr-based amorphous alloy liner.

As shown in Figure 9, under the same conditions of explosive charges, initiation mode and target materials, the jet DOP of Zr-based amorphous alloy liner was smaller than that of copper liner under the corresponding stand-off distances. The DOP of energetic liner was about 55.7% of the copper liner’s under the optimum stand-off distance (3.5 CD).

The entrance dimension of the penetration holes in the 1^st^ target column indicate that the penetration hole area of Zr-based amorphous alloy liner was larger than that of copper liner’s when the stand-off distance was 3.5 CD and 6.0 CD. Taking into account the pits produced by jet particles, the penetration was roughly distributed in a rectangular range of 90.0 mm × 55.7 mm (see Figure 7) when the stand-off distance was 22.0 CD, which was also larger than that of the copper liner’s. Therefore, it could be seen that the perforation area of Zr-based amorphous alloy liner was larger than that of the copper liner’s under the same stand-off distance. The jet perforation entrance area of the Zr-based amorphous alloy liner was about 2.8 times of the copper liner’s at the optimum stand-off distance (3.5 CD).

The perforation entrance size in the 2^nd^ target column (when the jet DOP reached 100mm) indicates that the advantage of the Zr-based amorphous alloy liner was no longer that obvious compared with that of the copper liner. That is to say, the retention ability of perforation area decreased obviously as the DOP decreased rapidly.

The reason for the “small penetration depth and large aperture” penetration performance of the energetic liner was that the energetic material released a lot of chemical energy in the process of high temperature and high speed jet penetrating the target plate. The energy mainly comes from two parts, one is the oxidation–reduction reaction between the metal elements and oxygen, the other is the combination reaction between the metal elements. The reaction products of Zr_63.5_Cu_12_Ni_10_Al_12.5_Ag_2_ amorphous alloy are mainly ZrO_2_, other components were Al_2_O_3_, NiO, CuO, Cu_2_O and a small amount of intermetallic compound Al_5_Ni_2_Zr. This is consistent with the phase analysis results of XRD combustion products given in reference [27]. The ablation and blackening could be seen at the enlarged hole of the target block, which also shows that the jet releases energy during the penetration process. Due to the energy releasing reaction of energetic material jet in the process of penetrating the target, its penetration ability was weaker than that of inert metal jet, but the internal explosive releasing energy of the energetic material jet could cause the metal target plate to burst and crack, which had a better aftereffect damage ability than that of the inert jet.

## 4. Conclusions

(1) Jet DOP of Zr-based amorphous alloy liners firstly increased slowly and then decreased sharply as the stand-off distance increased. The optimum stand-off distance was 3.5 CD and the corresponding maximum DOP was 122 mm against 45# steel plate, which was about 2.68 CD. The DOP had no obvious change when the stand-off distance was 2.0, 3.5 and 6.0 CD.

(2) The perforation area produced by jets of Zr-based amorphous alloy liners varied with the stand-off distance. It reached the maximum when the stand-off was 3.5 CD and the corresponding perforation diameter was about 42mm. The jet would disperse and granulate during the flight when the stand-off was 22.0 CD. Beside of a main penetration hole, which had a minimum diameter of 25 mm, several bigger shallow pits and smaller shallow spurting pits were produced on the target. The perforation area at 100 mm DOP against the 45# steel plate firstly increased and then decreased as the stand-off became larger. Specifically, the penetration area got a small value when the stand-off distance was 1.0 CD. No penetration hole formed under 22.0 CD condition. The perforation area were similar when the stand-off distance was 2.0, 3.5 and 6.0 CD, but the shape of hole was nearly circular when the stand-off distance was 3.5 CD.

(3) Under the same conditions, the jet DOP of Zr-based amorphous alloy liner was smaller than that of copper liner’s while the perforation area was the opposite. The former DOP was about 55.7% of the latter and the former perforation area was about 2.8 times of latter at the optimum stand-off distance (3.5 CD).

## Figures and Tables

**Table 1:** Results of Zr-based amorphous alloy liners during static explosion at the proving ground.

Serial Number	Stand-off/CD	Measured Value of Stand-off Distances/mm	Dimensions of Penetration Holes/mm (Horizontal Dimension × Vertical Dimension)				DOP		Remarks
			1^st^ Target Column (100 mm)	2^nd^ Target Column (50 mm)	3^rd^ Target Column (50 mm)	4^th^ Target Plate (150 mm)	mm	CD	
1	1.0	45.6	—	8 × 11	—	—	117	2.57	Unrecovered fragmentation in the 1^st^ target column; paper support tube
2	2.0	91.0	—	13 × 15	—	—	121	2.65	Unrecovered fragmentation in the 1^st^ target column; paper support tube
3	3.5	152.0	Entrance hole: φ42 Exit hole: φ15	φ14	—	—	122	2.68	The 1^st^ target column cracked into 2 blocks, gauging after recovered
4	6.0	260.5	Entrance hole: φ40 Exit hole: 14 × 9	15 × 8	—	—	120	2.63	
5	22.0	1000	φ25	—	—	—	37	0.81	

**Table 2:** Results of the copper liner during static explosion at the proving ground.

Serial Number	Stand-off/CD	Measured Value of Stand-off Distances/mm	Dimensions of Penetration Holes/mm (Horizontal Dimension × Vertical Dimension)				DOP		Remarks
			1^st^ Target Column (100 mm)	2^nd^ Target Column (50 mm)	3^rd^ Target Column (50 mm)	4^th^ Target Plate (150 mm)	mm	CD	
1	3.5	152.0	φ25	φ14	φ9	φ8	219	4.80	The 1^st^ target column cracked into 3 blocks, gauging after recovered
2	6.0	260.5	Entrance hole: φ24 Exit hole: 16 × 11	18 × 8	16 × 7	—	183	4.01	Hole plugging found in the 2^nd^ target column
3	22.0	1000	φ34	—	—	—	93	2.04	The 1^st^ target column cracked
